# Between Ethical Dialogue and Procedural Compliance: Greek Physicians’ Perspectives on Informed Consent and Sensitive Health Data

**DOI:** 10.7759/cureus.117632

**Published:** 2026-10-08

**Authors:** Emmanouil Zervos, Eleni Jelastopulu, Alexandra Skitsou, George Charalambous

**Affiliations:** 1 Department of Health Sciences, Frederick University, Nicosia, CYP; 2 Department of Public Health, University of Patras, Patras, GRC; 3 Department of Health Care Management/Department of Emergency Medicine, General Hospital of Athens "Ippokrateio", Athens, GRC; 4 Department of Health Care Management, Frederick University, Nicosia, CYP

**Keywords:** greece, informed consent, medical ethics, physicians, sensitive health data

## Abstract

This qualitative study explored perspectives on informed consent and sensitive health data among physicians recruited through the Medical Association of Rhodes. Ten physicians from varied specialities, places in Greece, and career stages participated in semi-structured interviews analysed thematically. Four themes described consent as ethical dialogue versus procedural documentation, communication and organisational barriers to patient understanding, uneven legal and ethical competence, and fragmented data governance alongside cautious optimism about digital tools. Meaningful consent depended on comprehensible communication, voluntary choice, protected consultation time, professional education, adaptable procedures, and secure data governance. The findings support institutionally coordinated practices that integrate patient autonomy, clinical relationships, and data protection within routine care.

## Introduction

Informed consent is a foundational requirement of ethically legitimate clinical practice. Its normative force rests on respect for autonomy, human dignity, and bodily integrity, yet its practical meaning extends beyond the completion of a signed form. Consent requires a person to receive relevant information in a comprehensible form, deliberate without coercion, ask questions, consider alternatives, and communicate a voluntary decision. International bioethical instruments embed this understanding in a wider human-rights framework. The UNESCO Universal Declaration on Bioethics and Human Rights links consent with autonomy, vulnerability, privacy, confidentiality, equality and social responsibility, while the Oviedo Convention establishes free and informed consent as the general rule for interventions in the health field and recognises the sensitivity of information concerning health [1,2]. These principles present consent and confidentiality as interrelated practices through which healthcare institutions recognise patients as rights-bearing persons.

Contemporary clinical environments complicate the enactment of these principles. Information must often be communicated in settings characterised by limited consultation time, high workload, emotional distress, differences in health literacy and variable access to interpretation or private space. The ethical quality of consent therefore depends on more than the physician’s intention to provide information. It is shaped by organisational arrangements, professional education, communication resources, and the patient’s social and informational circumstances. This broader perspective is consistent with accounts that treat autonomy as supported through relationships and institutional conditions rather than as an isolated act of individual choice [3,4]. A signature may record a decision, yet the moral validity of that decision depends on the communicative process that preceded it.

The expansion of digital health intensifies these concerns. Electronic health records, interoperable platforms, remote care, algorithmic inference, and secondary data use have widened the range of information that can reveal a person’s health status. Schneble et al. [5] argue that the boundary between health and non-health data is increasingly porous because combinations of apparently ordinary data can generate sensitive health-related inferences. In large-scale data ecosystems, consent obtained at a single point may provide limited control over subsequent linkage, reuse, and transfer. Saksena et al. [6] consequently propose a governance approach that combines consent with institutional accountability, privacy by design, and regulatory oversight. This approach shifts ethical attention from the individual’s agreement alone to the responsibilities of organisations that collect, store, and use health information.

European law reinforces the need to distinguish two forms of consent that are frequently conflated in clinical discourse. Informed consent to a medical intervention concerns the ethical and legal authorisation of treatment. Consent as a legal basis for personal-data processing operates within the General Data Protection Regulation (GDPR). Health data receive special protection under the GDPR, although processing for diagnosis, treatment, and the management of health services may rest on legal bases other than the data subject’s consent, subject to professional secrecy and appropriate safeguards [7]. In Greece, the relevant framework includes the ratification of the Oviedo Convention, the Code of Medical Ethics, and national measures implementing the GDPR [8-10]. Conceptual clarity matters because a patient’s agreement to treatment does not automatically settle every question regarding data collection, access, retention or secondary use.

Research on electronic consent illustrates both opportunities and ethical tensions. De Sutter et al. [11] identify regulatory variation across European jurisdictions, despite the capacity of electronic consent to support accessibility, documentation, and continuing information. Sahan et al. [12] similarly note that digital interfaces can improve presentation and traceability while also reproducing inequalities, reducing personal interaction or encouraging a checklist mentality. Zenker et al. [13], in work on broad consent for secondary research use, emphasise that the quality of consent depends on its integration with oversight, security, and organisational accountability. Public preferences also indicate that trust, transparent purposes, communication, and the identity of data recipients influence willingness to share health information [14-16]. These findings support an ethical model in which consent forms one component of a continuing relationship of accountability.

Empirical studies of clinical consent show a persistent gap between normative expectations and routine practice. In emergency surgical settings, patients and relatives may make decisions amid anxiety, urgency, and reduced capacity to process complex information [17]. Healthcare professionals identify low health literacy, language differences, complicated terminology, limited time, and the treatment of consent as legal protection as recurrent obstacles [18]. Procedure-specific forms can improve the consistency of information, although forms function most effectively when they support, rather than replace, dialogue [19]. These challenges are ethically significant because they distribute the practical ability to exercise autonomy unevenly. Patients with fewer educational, linguistic or social resources may receive the same document while experiencing a substantially different opportunity to understand and decide.

The Greek clinical context provides a valuable setting in which to examine these issues. It combines a developed legal framework and expanding digital health infrastructure with organisational pressures that may affect communication, privacy, and professional preparation. Published evidence has concentrated mainly on legal requirements, digital consent models, patient preferences, or specific procedural contexts*.* Less is known about how physicians working in Greece interpret the relationship between consent, communication, and sensitive data in everyday practice, or how they understand the institutional conditions that enable ethical implementation.

This study therefore explored physicians’ perceptions, experiences, and practices concerning informed consent and the management of sensitive medical data among physicians recruited through the Medical Association of Rhodes. Specifically, it addressed five objectives: to examine how physicians define meaningful consent, identify the barriers they associate with patient understanding, explore how they assess their legal and ethical preparation, describe their data-protection practices, and identify the reforms they consider most useful. By analysing these accounts through an empirical bioethics lens, the study aims to show how autonomy and confidentiality are enacted within concrete organisational settings and to identify implications that extend beyond a single national system.

## Materials and methods

Study design and ethical orientation

This study used an interpretive qualitative design based on semi-structured interviews. The design was selected because informed consent and health-data protection involve meanings, judgements, and practices that are experienced differently across clinical settings and professional careers. The analysis was informed by empirical bioethics, understood here as the integration of participants’ accounts with normative concerns surrounding autonomy, dignity, confidentiality, trust, and institutional responsibility. The purpose was to interpret how ethical principles were understood and enacted in practice rather than to measure their prevalence. Reporting was prepared with reference to the Consolidated Criteria for Reporting Qualitative Research checklist [20].

Setting and participants

Participants were physicians working in Greece who had practical experience of obtaining informed consent or handling sensitive medical information. Recruitment was facilitated through the Medical Association of Rhodes, which distributed the study invitation and research material to eligible specialist physicians in Greece. A convenience sampling strategy was used to recruit physicians from different specialities, work contexts, and levels of professional experience. Eligibility required current or recent clinical practice in Greece and direct familiarity with consent or medical-data processes. The final sample comprised ten physicians aged 32-74 years, with 7-40 years of professional experience. Nine participants were men, and one was a woman. The sample included one physiatrist, one general surgeon, two internists, one ophthalmologist, and five orthopaedic surgeons, providing representation from both medical and surgical specialities. Participants represented earlier and later career stages and worked across hospital and other clinical settings. The sample included physicians with varying levels of involvement in consent procedures, ranging from daily participation to occasional involvement.

Recruitment concluded after ten interviews. At that stage, the later interviews largely repeated the principal codes and did not add substantively different meanings to the developing thematic structure. In qualitative inquiry, sample adequacy is assessed in relation to the specificity of the sample, the study aim, the quality of dialogue and the analytic strategy rather than through a numerical threshold alone [21]. The present sample was considered adequate for an exploratory study focused on a defined professional group, while the claims were framed as contextual and interpretive.

Data collection

Data were generated through individual semi-structured interviews. The interview guide was adapted from the open-access Research Staff Survey developed by O’Sullivan et al. [22] and published as supplementary material to their 2021 Trials study. The source article and its supplementary survey are openly available under a Creative Commons Attribution 4.0 International licence. Relevant survey domains were reformulated as open-ended prompts for a qualitative interview format and supplemented with questions addressing the Greek legal, ethical, and health-data governance context. Semi-structured interviewing provided a common set of domains while allowing participants to elaborate on experiences, examples, and professional concerns in their own terms [23].

The interview guide covered five areas. The first concerned demographic and professional characteristics, including age, experience, work setting, and frequency of involvement in consent. The second explored participants’ definitions of informed consent, the information they considered necessary before a medical or surgical intervention, perceptions of patient understanding, communication barriers, time devoted to information, and responses to refusal. The third addressed familiarity with the legal and ethical framework and previous training. The fourth focused on the collection, storage, access, and protection of sensitive medical data, including electronic record systems and patient information about data handling. The fifth examined trust, professional responsibility, training needs, and the potential role of digital tools (see Appendices). All five core domains were addressed with every participant, while follow-up probing varied according to the detail of participants’ responses and their professional experience. One interview item explicitly invited participants to reflect on the relationship between dialogue and procedural documentation; during analysis, this item was treated as a sensitising prompt rather than as independent evidence that this contrast arose spontaneously.

Interviews were conducted in person in locations that supported privacy. Interviews lasted approximately 15-20 minutes, with variation reflecting the amount of detail participants provided and the extent of follow-up probing, while the same core topics were retained across interviews. With explicit permission, interviews were audio-recorded and subsequently transcribed verbatim. Identifying details were removed from the working transcripts, and participant codes P1-P10 were used in the analysis and reporting. The research material was stored on encrypted devices with access limited to the research team. Participants received information about the study purpose, voluntary participation, confidentiality, audio recording, the planned use of anonymised extracts and the right to withdraw.

Data analysis

The transcripts were analysed using thematic analysis following Braun and Clarke’s six-phase approach [24]. Analysis began with repeated reading of the transcripts and comparison of each transcript with its recording for accuracy. Initial codes were then developed across the dataset. Coding combined an inductive orientation toward participants’ language and concerns with theoretically informed attention to autonomy, communication, confidentiality, trust, and institutional responsibility.

Related codes were grouped into candidate themes. The developing themes were reviewed against the coded extracts and the full set of transcripts, with attention to internal coherence, distinction between themes and variation across participants. Theme names and definitions were refined to express the central organising idea of each pattern. The final analysis produced four themes that connected participants’ practical accounts with the study’s bioethical questions. The participant-profile material was treated descriptively and was not classified as an analytic theme.

Trustworthiness and reflexivity

Several procedures supported analytic transparency. The research team maintained an organised record of transcripts, codes, theme development and revisions. Repeated movement between the developing interpretation and the primary material helped preserve the connection between claims and participant accounts. Comparisons across participants were used to identify convergence and variation, including views that diverged from the dominant pattern. Reflexive consideration was given to the influence of the researchers’ clinical, legal, and health-services perspectives on the interpretation of the data. The analysis sought interpretive credibility and a clear audit trail rather than statistical reliability [25].

Ethical approval

All participants provided written informed consent before participation. The study procedures were conducted in accordance with the principles of voluntary participation, confidentiality, anonymity, and lawful processing of personal data under Regulation EU 2016/679 GDPR. The Medical Association of Rhodes authorised the distribution of the study invitation and research material to eligible specialist physicians who were members of the Association. This authorisation is documented in the official certificate issued by the Medical Association of Rhodes, Protocol No. 608/2026, dated 31 August 2026, which confirms that the research material had been distributed to eligible physicians on 7 April 2025. Participation remained entirely voluntary, and the Association had no involvement in data collection, analysis, or interpretation.

## Results

Participant characteristics

The ten participants represented a broad age and experience range. Three reported daily involvement in consent procedures, four described very frequent or weekly involvement, and three reported occasional or monthly involvement. Formal education specifically addressing informed consent was uncommon. Only one participant recalled relevant exposure outside Greece, and this was described as general rather than comprehensive. Consent discussions occurred in consulting rooms, during preoperative assessment, and at the bedside. These settings differed in the time and privacy available for communication. Participant characteristics are summarised in Table 1.

**Table 1:** Participant characteristics

Characteristic	Description
Sample	10 physicians working in Greece
Gender	9 men and 1 woman
Age	32-74 years
Professional experience	7-40 years
Involvement in informed consent	3 daily; 4 very frequent or weekly; 3 occasional or monthly
Formal training in informed consent	1 participant reported limited previous exposure; 9 reported none
Clinical context	Hospital and other clinical settings
Specialities	1 physiatrist, 1 general surgeon, 2 internists, 1 ophthalmologist, and 5 orthopedic surgeons

Theme 1: Consent as ethical dialogue and procedural documentation

Participants generally defined informed consent in ethically substantive terms. They referred to complete and understandable information, voluntary agreement, and the patient’s opportunity to decide after learning about the intervention. Several emphasised the nature and purpose of the procedure, expected benefits, possible complications, alternative options, and the right to refuse. P3 described consent as “full information on the way the procedure is performed and the complications,” while P8 extended the definition to research contexts through “information on study stages, obligations, timeline, benefit and adverse events.” These accounts indicate that participants recognised consent as a process rather than a signature alone.

At the same time, a broader pattern across the interviews indicated a recurrent distance between this normative understanding and routine implementation. This pattern was reflected in descriptions of time-limited exchanges, variable patient understanding, reliance on standard forms, and pressure to complete documentation within busy clinical workflows. P4 estimated that “forty to sixty percent do not understand; consent ends up being formal.” This estimate is presented as one participant’s perception rather than as a prevalence measure. It illustrates, rather than establishes, the wider theme identified through convergent accounts of communication constraints, documentation practices, and organisational pressures. Physicians could articulate the ethical content of consent while working in circumstances that could encourage procedural completion.

The distinction between dialogue and documentation also appeared in accounts of refusal. Participants consistently endorsed respect for a competent patient’s decision. P3 recalled that when consent was withheld, “the procedure did not proceed,” and P4 referred to “absolute respect for refusal.” Several participants described offering further explanation in simpler terms before accepting the final decision. P7 explained that the patient was informed that “without consent the procedure does not proceed.” These responses suggest that refusal was recognised as a genuine expression of autonomy rather than merely an obstacle to care.

Participants also associated inadequate consent with legal and relational consequences. Some were aware of claims arising from limited disclosure of complications or alternatives. P3 referred to “legal action for compensation,” while P4 recalled an incident involving incomplete information about available options. Such examples reinforced the defensive dimension of consent. For some physicians, the possibility of litigation increased attention to documentation, yet it could also strengthen a view of consent as institutional protection rather than shared decision-making.

Theme 2: Understanding is shaped by communication and organisational conditions

Physicians described patient understanding as highly variable. Educational background and health literacy were frequently mentioned, together with familiarity with medical language and the emotional state of the patient. P2 characterised understanding as “very low” in many encounters. P8 offered a more conditional assessment, stating that “it depends on educational level; an effort is made to support understanding.” The contrast between these responses indicates that physicians recognised both patient-related variation and their own communicative responsibility.

Anxiety, fear of complications, family pressure and misinformation were prominent barriers. Participants described patients who arrived with information from online or social sources that complicated the clinical discussion. P10 identified “patient or family anxiety, time pressure, misinformation” as interacting difficulties. Language differences and cultural or religious beliefs were also mentioned. These factors could influence the questions patients asked, the meanings they attached to risk and their willingness to disclose uncertainty.

The physical environment affected confidentiality and concentration. P8 described situations involving “prejudice, limited time, and consent obtained in a space with other patients.” Bedside communication in shared areas created a direct tension between the need to provide timely information and the ethical requirement for privacy. Participants viewed a quiet and private setting as particularly important when discussing serious diagnoses, complex procedures, or sensitive personal information.

Time was the most consistent organisational constraint. Typical discussions lasted approximately 10-15 minutes, with longer conversations for complex cases or when patients asked additional questions. P9 reported “about fifteen minutes,” while P10 described “five to ten minutes, more time if there are questions.” Participants linked shorter encounters with omissions, reduced opportunity for clarification, and greater reliance on standard forms. Adequate time, by contrast, enabled the physician to adapt language, check understanding, and address concerns.

Workload intensified these pressures. Participants described busy hospital environments in which the ethical intention to communicate carefully competed with service demands. Consent quality therefore depended partly on staffing, workflow, and the location of the encounter. The accounts positioned meaningful consent as an organisational achievement rather than a task that could be secured by individual professionalism alone.

Theme 3: Legal and ethical competence as an institutional gap

Knowledge of the legal framework varied considerably. A small number of participants described themselves as reasonably familiar with the duty to obtain consent, while most used expressions such as “very little,” “a little,” or “not enough.” P4 described informed consent as “a basic legal and ethical principle,” whereas P1 answered “very little” when asked about familiarity with the legal obligation. The variation was especially notable because all participants encountered consent in clinical work.

Views on whether the legal framework protected both patients and physicians were equally diverse. Some regarded the framework as adequate. Others believed that it primarily protected patients, or that practical uncertainty left both parties exposed. P2 stated, “It protects the patient more, not the doctor,” while P7 answered that it protected “no one.” Two participants said that they did not know. These responses may reflect differences in legal knowledge, prior experience, and the perceived accessibility of institutional guidance.

Training emerged as the clearest shared concern. Almost every participant reported an absence of structured legal or institutional education on informed consent. The one participant who recalled general exposure regarded it as insufficient for practice. The brevity and consistency of responses such as “No” conveyed a marked reliance on experiential learning, local custom, and individual initiative.

Participants linked this educational gap with inconsistency. Without shared training, physicians developed their own approaches to risk disclosure, documentation, refusal, and data handling. The resulting variation was understood as both a professional and institutional issue. Participants proposed mandatory seminars, continuing education, and accessible guidance that integrated ethical reasoning with practical legal knowledge.

Theme 4: Fragmented data governance and cautious technological optimism

Practices for protecting sensitive medical data differed across settings. Some participants described password-protected electronic systems, role-based or restricted access, secure storage, and anonymisation. Others worked with broader team access, handwritten files, or basic local safeguards. The diversity reflected differences in institutional infrastructure, cost, and organisational maturity rather than a single professional norm.

Patient information about data storage and use was also inconsistent. Verbal explanation was the most common approach, while written information was used less regularly. Participants recognised confidentiality as important, yet several expressed uncertainty about whether patients understood who could access their records, how long information was retained, or how data might circulate within a clinical team. The ethical issue therefore extended beyond technical security to transparency about information practices.

Electronic records were viewed as capable of improving traceability, continuity, and access control. At the same time, participants raised concerns about cost, unequal implementation, and the possibility that technology could become a substitute for conversation. Digital signatures, online guidance, and multimedia information were generally welcomed as supports for standardisation and comprehension. The preferred model combined technology with personal explanation and opportunities for questions.

The reform proposals converged around four elements. Participants called for systematic education in informed consent, medical law and the GDPR; standardised but understandable consent materials; protected time and appropriate space for dialogue; and stronger technical procedures for data access and storage. Trust linked these recommendations. Participants regarded transparency, respectful communication, and secure handling of information as mutually reinforcing conditions of the physician-patient relationship. The final themes, subthemes, and illustrative evidence are summarised in Table 2.

**Table 2:** Final themes, subthemes, and illustrative evidence Note: Participant codes P1-P10 were used in the analysis and reporting. Participant codes preserve anonymity.

Theme	Subthemes	Illustrative evidence
1. Consent as ethical dialogue and procedural documentation	Comprehensible information; voluntary decision; procedural formality; respect for refusal; defensive documentation	P3: “Full information on the way the procedure is performed and the complications.” P4: “Forty to sixty percent do not understand; consent ends up being formal.” P7: “Without consent the procedure does not proceed.” Across accounts, this theme was also supported by descriptions of time pressure, reliance on standard forms, and defensive documentation.
2. Understanding shaped by communication and organisational conditions	Health literacy; anxiety; language and cultural barriers; misinformation; workload; time; privacy	P2: “Very low level of understanding.” P8: “It depends on educational level.” P10: “Patient or family anxiety, time pressure, misinformation.”
3. Legal and ethical competence as an institutional gap	Variable familiarity; uncertainty about legal protection; limited formal education; reliance on experiential learning	P1: “Very little.” P2: “It protects the patient more, not the doctor.” Most participants answered “No” when asked about structured training.
4. Fragmented data governance and cautious technological optimism	Uneven safeguards; inconsistent patient information; differences in electronic-record use; digital tools as supportive resources	Reported practices ranged from password protection, restricted access and anonymisation to handwritten records and basic local safeguards. Participants favoured digital support that preserved interpersonal dialogue.

## Discussion

This interpretation aligns with longstanding critiques of highly individualised and form-based models of consent. Manson and O’Neill [4] argue that informed consent is fundamentally communicative and depends on relevant, intelligible, and trustworthy information. Beauchamp and Childress [3] similarly treat intentionality, understanding, and freedom from controlling influence as core conditions of autonomous action. The participants’ accounts suggest that these conditions were recognised conceptually but were variably realised in practice. The gap did not arise simply from a lack of ethical commitment. It emerged from the interaction of workload, privacy limitations, patient anxiety, language, health literacy, and inconsistent institutional guidance.

Participants’ accounts of incomplete or variable patient understanding resemble evidence from surgical and hospital contexts. Negash et al. [18] identified medical terminology, anxiety, time limitations, and low health literacy as barriers reported by patients and professionals. Kituuka et al. [17] showed how urgency and emotional distress constrain decision-making in emergency surgery. The present study adds a structural dimension by showing that physicians themselves located these barriers within the organisation of care. A patient may receive the same written form as another patient while having less time, privacy, linguistic support, or confidence to ask questions. Formal equality of documentation can therefore coexist with substantive inequality in the capacity to decide.

This point has significance for the study of ethical behaviour in healthcare. The UNESCO declaration presents autonomy, consent, vulnerability, privacy, and equality as interconnected principles rather than isolated requirements [2]. The present findings demonstrate why this integration matters in a high-income European setting. Vulnerability may be produced situationally through anxiety, limited health literacy, crowded wards, or institutional time pressure. Ethical practice consequently requires attention to the social and organisational distribution of communicative opportunity. Consent becomes more equitable when patients receive information adapted to their needs, adequate time for deliberation, and a private setting in which uncertainty can be expressed.

The mixed role of legal protection deserves similar attention. Participants’ awareness of complaints and litigation encouraged careful documentation, but it also supported a defensive understanding of consent. When consent is framed primarily as protection against liability, the ethical objective can shift from enabling patient judgement to demonstrating procedural compliance. Procedure-specific forms may improve consistency and recall, as Norvill et al. [19] report, yet their value depends on integration with dialogue. Standardisation should therefore define a reliable minimum of information while preserving adaptation to the patient’s clinical condition, preferences, literacy, and questions.

Respect for refusal was one of the strongest ethically positive findings. Participants described further explanation, simplification, and eventual acceptance of the patient’s decision. This suggests that autonomy was not understood solely as agreement with the proposed intervention. The right to decline remained visible in physicians’ accounts, consistent with the Oviedo Convention and UNESCO framework [1,2]. Future training can build on this foundation by addressing difficult cases involving fluctuating capacity, emergencies, family influence, communication barriers, and disagreement about risk.

The reported lack of structured training is especially important. Most participants relied on professional experience rather than formal preparation in consent, medical law, or data protection. Variation in familiarity with the legal framework was accompanied by uncertainty about whether existing rules protected patients, physicians, or both. These findings indicate that legal and ethical competence should be treated as an institutional responsibility. A system that establishes detailed obligations while leaving clinicians to interpret them through informal learning creates predictable inconsistency. Continuing education should connect normative principles with realistic clinical scenarios, documentation standards, and communication techniques.

Training also needs to clarify the relationship between treatment consent and data protection. The two were closely associated in participants’ accounts, yet they are legally and ethically distinct. Informed consent authorises a medical intervention, whereas the GDPR regulates the processing of personal data through a set of legal bases, duties and safeguards. In healthcare, the processing of health data for diagnosis, treatment and service management may be lawful without relying on GDPR consent, provided that the relevant conditions and professional confidentiality protections apply [7]. Clear education on this distinction can reduce the tendency to treat a single consent form as universal authorisation for treatment and every subsequent data use.

The heterogeneity of data-protection practices suggests that confidentiality was often operationalised through local infrastructure. Passwords, restricted access and encrypted storage were available in some settings, while handwritten records, broad team access, and informal explanation remained present elsewhere. This pattern corresponds with wider concerns about the governance of increasingly interconnected health information. Schneble et al. [5] show that health sensitivity can arise through data combination and inference, while Saksena et al. [6] argue that individual consent requires support from privacy-by-design and accountable institutions. The present findings reinforce the view that confidentiality depends on system architecture, access controls, staff preparation, and transparent governance as much as on personal discretion.

Public attitudes toward data sharing further illuminate the importance of trust. Systematic reviews indicate that willingness to share health data varies according to purpose, recipient, perceived benefit, and confidence in safeguards, with lower acceptance when commercial involvement or future use is unclear [14,15]. Müller et al. [16] likewise identify continuing communication and the return of information as important to public preferences. Physicians in the present study connected secure data handling with the quality of the therapeutic relationship. This connection suggests that privacy practices communicate institutional respect. A technically secure system accompanied by opaque explanations may still weaken trust, while transparent communication without adequate safeguards leaves confidentiality exposed.

Participants’ cautious optimism regarding technology is consistent with the emerging literature on electronic consent. Digital platforms can present information in layers, use multimedia, document updates, and support remote access. De Sutter et al. [11] describe these advantages alongside regulatory complexity, and Sahan et al. [12] emphasise the need to preserve meaningful interaction and avoid digital exclusion. The physicians’ preferred approach was similarly hybrid. Technology was seen as useful for standardisation and comprehension, while personal discussion remained central. This balance is ethically appropriate because technological efficiency and relational care serve different functions.

The findings support several practical priorities. First, healthcare organisations can allocate protected time and private space for high-stakes consent conversations. Second, national or institutional templates can establish core information while allowing procedure-specific and patient-specific adaptation. Third, communication aids can use plain language, visual material and multilingual resources, with a teach-back process to assess understanding. Fourth, continuing professional education can integrate bioethics, medical law, health literacy and data governance. Fifth, electronic systems can apply role-based access, audit trails, secure authentication and clear retention policies. Patient representatives can contribute to the design of forms and digital tools so that standardisation reflects users’ informational needs.

The specific contribution of this study is not the claim that documentation uniformly replaces dialogue, but the identification of organisational conditions that can shift consent along a continuum between relational communication and procedural completion. Meaningful consent emerges here as an institutional practice of autonomy, shaped by physician communication, the patient’s opportunity to understand, available time and privacy, organisational procedures, and information-system security. This interpretation places ethical responsibility within both professional practice and the structures that enable clinicians and patients to exercise it.

Strengths and limitations

The study brought together physicians with a wide age range, different career stages, and varying frequency of involvement in consent. The qualitative design generated detailed accounts of ethical tensions that may remain hidden in surveys or legal analyses. The integration of consent and health-data governance also reflects the way these issues intersect in contemporary clinical practice.

The findings should be interpreted in relation to the convenience sample of ten physicians, the predominance of male participants, the use of a single recruitment channel through the Medical Association of Rhodes, and the limited range of clinical settings represented. The available participant description does not provide a sufficiently granular breakdown by public versus private sector or by hospital versus primary-care practice, which limits assessment of transferability across organisational contexts. The study examined professional accounts rather than observed encounters, patient experiences or institutional records, and self-report may reflect participants’ interpretations of desirable practice as well as routine behaviour. The exploratory design supports conceptual and contextual transferability rather than population-level generalisation. Future research could include patients, nurses, data-protection officers and administrators, compare public and private settings and levels of care, and combine interviews with observation or document analysis.

## Conclusions

This study indicates that physicians in this exploratory sample recognised informed consent as an ethical process grounded in information, voluntariness, and respect for refusal. Their accounts also suggest that clinical workload, limited privacy, patient anxiety, differences in health literacy, uneven legal preparation, and fragmented data infrastructures can redirect that process toward procedural completion. The ethical challenge therefore concerns the conditions under which consent is practised, rather than the existence of a form alone.

Consent and confidentiality should be supported through coordinated institutional measures. Standardised and adaptable information resources can improve consistency, while protected time, private communication, teach-back, and multilingual support can strengthen understanding. Continuing education can clarify the ethical and legal distinction between consent to treatment and the lawful processing of health data. Secure digital systems can reinforce accountability through controlled access, auditability, and transparent explanations to patients. These exploratory accounts have wider relevance for research on ethical behaviour in healthcare because they illustrate how formal protections are mediated by organisational conditions. Formal legal protections acquire practical meaning through organisational capacity, professional competence, and trustworthy relationships. Healthcare systems that treat autonomy and privacy as shared institutional responsibilities are better positioned to transform consent from a compliance event into a genuine practice of patient participation and trust.

## Appendices

Semi-structured interview questions organized into 5 thematic areas, suitable for physicians.

Thematic Area 1: Demographic characteristics of respondents

1. What is your gender?

2. At which hospital or clinic do you work?

3. What is your age?

4. What is the highest level of education you have completed (Bachelor’s, Master’s, or PhD)?

5. How many years of professional experience do you have in the medical field?

6. How often are you involved in the informed consent process?

7. What is your experience in managing informed consent discussions with patients and/or their families?

8. Have you attended specialized training or seminars on informed consent?

9. If yes, what were the main topics covered in this training, and do you believe the training you received is adequate for your daily practice?

10. At what stage of the treatment process and in what setting do you usually obtain informed consent?

Thematic Area 2: Perception and importance of informed consent

11. How do you define the concept of "informed consent" in your daily practice? 12.            What do you consider to be the key elements that should be included when informing a patient prior to a medical or surgical procedure?

13.       To what extent do you believe patients understand the information provided to them during the consent process?

14.       Are there specific difficulties or obstacles you have observed in communicating with patients regarding informed consent?

15.       How much time do you usually devote to the information-giving process, and how does this affect the quality of the consent?

16.       Have you encountered cases where a patient refused to give consent? How did you handle the situation?

17.       Have you been involved in, or are you aware of, incidents where the absence of adequate consent led to consequences (legal or otherwise)?

Thematic Area 3: Legal and ethical framework

18.       How familiar are you with the legal obligation regarding informed consent in Greece?

19.       Do you believe the current legal framework adequately protects both patients and physicians?

20.       Have you received legal or institutional training regarding the implementation of informed consent? If so, do you consider it to have been adequate?

Thematic Area 4: Collection and protection of sensitive medical data

21.       How do you ensure the confidentiality of the medical data collected from patients?

22.       What are the most common challenges you face regarding the management of sensitive data in clinical practice?

23.       How do you inform patients about where and how their data will be stored?

24.       Do you use electronic recording systems? If so, what security measures are implemented to protect personal data?

25.       Have you received any formal training regarding the General Data Protection Regulation (GDPR) and its application in medical practice?

Thematic Area 5: Ethics, trust, and prospects for improvement

26.       What is your personal stance on the informed consent process: do you view it as a formal obligation or as a substantive ethical duty?

27.       What role do workload or time pressure play in the quality of the consent process you conduct?

28.       Do you believe that physicians in Greece are adequately prepared to implement the principles of informed consent and data protection?

29.       What measures would you propose to improve the patient information and consent process? What would you consider to constitute "good practice" regarding informed consent in medical practice?

30.       Do you believe that the use of new technologies (e.g., multimedia information tools, digital signatures) can enhance transparency and understanding in the consent process?
